# Selenium and cancer – what have we learned from epidemiology and molecular epidemiology studies?

**DOI:** 10.1186/1897-4287-13-S1-A12

**Published:** 2015-09-09

**Authors:** Ewa Jablonska, Jolanta Gromadzinska, Wojciech Wasowicz

**Affiliations:** 1 Department of Toxicology and Carcinogenesis Nofer Institute of Occupational Medicine, Lodz, Poland

## 

Selenium (Se) is an essential trace element with a wide spectrum of biological activity and as a potential anticancer agent, it has gained a lot of scientific attention. Since the first hypothesis concerning chemopreventive properties of selenium was formulated (in 1969), numerous studies on Se and cancer, including human randomized controlled trials, have been conducted. Whereas studies *in vivo* indicated that Se supplementation protected animals from chemically or biologically induced cancer, epidemiological studies generated contradictory data, and it seems that the relationship between selenium and cancer prevention in humans is complex. In the light of recent epidemiological data suggesting that long term supplementation with Se does not prevent prostate cancer in men and may increase the risk of diabetes in individuals with high selenium status, the use of Se in terms of cancer prevention is not recommended for the general population. It is considered that the beneficial effects of Se are probably limited only to the undernourished populations and they are strictly related to dose (in a U-shaped manner) and the chemical form of this trace element.

Functions of Se in human organism are mainly associated with the presence of selenoproteins. These are proteins containing Se in the form of selenocysteine (Sec), the 21^st^ amino acid, which due to its own codon in mRNA, is incorporated into the polypeptide chain during protein biosynthesis. So far more than 25 human selenoproteins have been identified, including those with important enzymatic activity (glutathione peroxidases, thioredoxin reductases and iodothyronindeiodinases). Notably, functions of some selenoproteins are still not recognized. It has been recently shown that polymorphic variants of selenoprotein encoding genes are associated with the altered cancer risk at different sites (lung, prostate, colon, breast, and bladder). Such associations have already been found for genetic polymorphism of cytosolic glutathione peroxidase (GPx1), phospholipid glutathione peroxidase (GPx4), 15 kDaselenoprotein (Sep15), thioredoxin reductase 1 (TrxR1) and selenoprotein P (Sepp1). Our study also indicated that genetic polymorphism of Sep15 modified the risk of lung cancer in the interaction with selenium status. Similar type of interactions was observed recently by other authors in prostate cancer individuals, in which the risk was modified by Se status and genetic polymorphisms of three selenoproteins: SelK, TrxR1 and TrxR2. These two studies (on lung and prostate cancer) indicate that genetic susceptibility associated with polymorphic variants of selenoprotein encoding genes, may be another, apart from dose a chemical form, factor that affects biological activity of selenium in humans.

